# Mechanisms Underlying Alcohol-Approach Action Tendencies: The Role of Emotional Primes and Drinking Motives

**DOI:** 10.3389/fpsyt.2014.00044

**Published:** 2014-05-06

**Authors:** Janna Cousijn, Maartje Luijten, Reinout W. Wiers

**Affiliations:** ^1^ADAPT-Lab, Department of Psychology, University of Amsterdam, Amsterdam, Netherlands; ^2^Department of Developmental and Experimental Psychology, Utrecht University, Utrecht, Netherlands; ^3^Institute of Psychology, Erasmus University Rotterdam, Rotterdam, Netherlands; ^4^Behavioural Science Institute, Radboud University Nijmegen, Nijmegen, Netherlands

**Keywords:** alcohol abuse, approach bias, approach avoidance task, emotional primes, drinking motives

## Abstract

The tendency to approach alcohol-related stimuli is known as the alcohol-approach bias and has been related to heavy alcohol use. It is currently unknown whether the alcohol-approach bias is more pronounced after emotional priming. The main aim of this study was to investigate whether positive and negative emotional primes would modulate the alcohol-approach bias. For this purpose, a new contextual emotional prime-approach avoidance task was developed, containing both negative and positive emotional primes. Explicit coping drinking motives were expected to be related to an increased alcohol-approach bias after negative primes. Results of 65 heavy and 50 occasional drinkers showed that the alcohol-approach bias was increased in both groups during negative emotional priming. This appeared to be due to slower alcohol avoidance rather than faster alcohol approach. This change in alcohol-approach bias was positively related to explicit enhancement drinking motives and negatively related to alcohol use-related problems. A stronger alcohol-approach bias in heavy compared to occasional drinkers could not be replicated here, and coping drinking motives were not related to the alcohol-approach bias in any of the emotional contexts. The current findings suggest that both occasional and heavy drinkers have a selective difficulty to avoid alcohol-related cues in a negative emotional context. Negative reinforcement may therefore be involved in different types of drinking patterns. The influence of emotional primes on alcohol-related action tendencies may become smaller when alcohol use becomes more problematic, which is in line with habit accounts of addiction.

## Introduction

Researchers have distinguished between implicit or relatively automatic cognitions on the one hand and explicit cognitive processes on the other. Implicit cognitions are spontaneously activated and require little resources, whereas explicit cognitions are more related to conscious decision making and rational weighing of pros and cons of behavioral options (1–4). Many studies have now demonstrated that implicit and explicit cognitions predict unique variance in alcohol use and problems [for meta-analyses see Ref. (5, 6)]. Furthermore, a number of studies have demonstrated that individual differences in executive control capacity moderate the relative balance between implicit and explicit cognitions, with implicit cognitions having a stronger influence on behavior in individuals with relatively weak executive control [(7–10); for review see Ref. (11)]. Implicit cognitions including attentional bias, approach bias, and implicit memory associations toward alcohol-related cues seem to have an important role in the development of alcohol-related problems (3). It is important to note, however, that inconsistent findings have also been reported (5, 6). Implicit and explicit cognitions have recently been placed on a continuum, with implicit cognitions representing initial information processing that can lead to action and explicit cognitions representing more elaborate information processing (11–13).

Explicit cognitions, including drinking motives, have traditionally been subdivided into three broad classes: positive reinforcement (expected pleasurable outcomes), negative reinforcement (expected relief of aversive states), and negative expected outcomes of continued alcohol use (14, 15). Research on explicit cognitions has consistently demonstrated that negative reinforcement plays an important role in the development of alcohol-related problems (14, 16–18). Perhaps surprisingly, research into implicit cognitions has primarily focused on automatically activated positive reinforcement cognitions (3). Only some studies have attempted to assess automatically activated negative reinforcement cognitions [i.e., (19–22)], with limited success.

Both coping and enhancement motives to drink alcohol are explicit cognitions common among heavy alcohol-drinking populations, although coping motives have been more strongly linked to problem drinking (23–26). These explicit drinking cognitions have been found to be sensitive to mood manipulations (27). Whereas high coping drinkers were found to report increased relief expectancies in a negative mood state, enhancement drinkers expected increased rewarding effects of alcohol after a positive mood induction.

With regard to implicit cognitions, negative (but not positive) mood priming has been found to implicitly activate alcohol-related concepts (i.e., beer) in heavy drinkers, covarying with alcohol problem levels (28). Negative mood inductions have also been found to increase implicit processes such as craving in alcohol-dependent patients (29), and a negative emotional state has been linked to beer consumption in young drinkers (30) and relapse (31). Interestingly, a recent study demonstrated that a negative mood induction implicitly activated alcohol-approach memory associations in regular alcohol drinkers with high, but not low coping motives (20). In line with this finding, individuals who reported drinking to cope with anxiety showed an increased attentional bias for alcohol-related words after an anxious mood induction, whereas drinkers high on enhancement motives showed increased attentional bias after a positive mood induction (32).

To summarize, previous findings suggest that a specific emotional state may influence the expression of implicit cognitions in heavy drinkers and that this relationship may depend on explicit drinking motives. The presentation of negative pictures (negative emotional primes) may then act as an internal cue, triggering alcohol-approach tendencies in heavy drinkers with strong coping motives. The presentation of positive pictures (positive emotional primes) may specifically activate alcohol-approach tendencies in heavy drinkers with strong enhancement motives. The effect of emotional primes (as a minimal emotional context) on alcohol approach and avoidance tendencies has not as yet been investigated.

For the purpose of the current study, a new emotional prime approach avoidance task (EP-AAT) was developed. The EP-AAT includes neutral appetitive (non-alcoholic drinks) as well as negative and positive primes to measure automatically activated approach and avoidance action tendencies after different emotional primes. Within the EP-AAT, a primed emotional context was operationalized by presenting alcohol pictures together with negative, positive, and non-alcoholic drink pictures in three separate blocks. We hereby followed a method developed by Mitchell et al. (33), to assess emotional context effects in a different task (a Go/NoGo task). Both heavy and occasional drinkers completed the task allowing us to investigate whether the presence of emotional primes influenced the expression of an alcohol-approach bias in heavy drinkers. As in the original substance AAT (34, 35), participants were instructed to use a joystick and react to the format of the picture (irrespective of the contents). Previous research using different varieties of this task has shown that heavy drinkers have an approach bias for alcohol (35) and heavy cannabis smokers an approach bias for cannabis-related materials (34). This is believed to reflect a sensitized response to cues associated with substance use (3, 34, 36). In addition, experimental studies have repeatedly shown a relationship between a substance-specific approach bias and actual substance use [for a review see Ref. (37)].

To the best of our knowledge, previous studies have not investigated the relationship between emotional primes and alcohol-approach action tendencies. Besides investigating the effects of different emotional primes, we also investigated the moderating role that coping and enhancement drinking motives have on the relationship between alcohol-approach bias and emotional primes. Across the appetitive, positive, and negative primed contexts, heavy drinkers were hypothesized to have a stronger alcohol-approach bias compared to occasional drinkers. Given the previously observed relationship between negative mood induction, coping motives, and activation of implicit alcohol-approach memory associations (20), coping drinking motives were expected to be related to an increased alcohol-approach bias after negative primes, compared with the other blocks. Enhancement drinking motives were expected to be related to an increased alcohol-approach bias in the positive compared to the appetitive context.

## Materials and Methods

### Participants

One hundred and forty-eight alcohol-drinking students were recruited thought advertisements on the internet and on the university campus. Participants were required to drink alcohol at least once per month. Thirty-two participants were excluded from analyses because of missing data (*N* = 12) and indications of psychiatric problems (*N* = 20). The remaining participants (*N* = 116) were classified as heavy (*N* = 66) or occasional drinkers (*N* = 50) based on Alcohol Use Disorder Identification Test [AUDIT; (38)] scores. In line with previous research (38), a cut-off score of 8 was used to classify occasional and heavy drinkers in the current study. The current study was approved by the ethics committee of the Institute of Psychology of the Erasmus University Rotterdam.

### Questionnaires

The AUDIT (38) was used to assess alcohol-related problems during the past 6 months. The AUDIT consists of 10 items assessing consumption and alcohol-related problems. Scores range between 0 and 40, with a cut-off score of 8 for hazardous drinking (38). To estimate recent quantitative alcohol use, an alcohol timeline follow back [TLFB; (39)] was used assessing alcohol use during the 10 days prior to participation. Using a calendar, participants indicated the number of standard drinks they consumed per day, starting with a day before and going back 10 days earlier. Furthermore, demographics (age, sex, level of education) and a detailed history of alcohol, nicotine, and illicit substance use were obtained, including onset, frequency, and quantity.

Craving for alcohol was measured with the Dutch version of the Desires for Alcohol Questionnaire [DAQ; Dutch version (40)] at the beginning (pre-test) and end (post-test) of the test session. The DAQ consisted of 14 items that were rated on a 7-point Likert scale ranging from “not at all” to “strongly agree” (41). Item scores were summed across dimensions to derive a single pre-test and post-test craving score per participant.

Coping and enhancement drinking motives were measured with the Drinking Motives Questionnaire – Revised [DMQ-R; (42)]. The DMQ-R measures the frequency of four distinct motives to drink alcohol: enhancement, coping, social, and conformity motives. It consists of 20 statements representing these different motives, and participants are asked to indicate how often they drank alcohol for a specific motive on a 5-point Likert scale ranging from “rarely/never” to “almost always/always.”

### Emotional prime approach avoidance task

**Figure 1:**
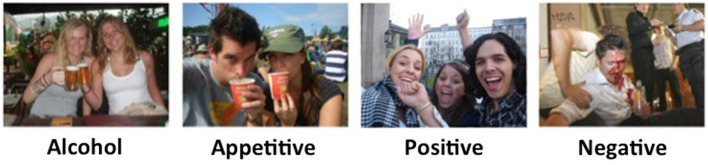
**Examples of pictures in the alcohol, appetitive, positive, and negative category**.

To measure approach and avoidance action tendencies toward alcohol stimuli in an appetitive, positive, and negative contexts, the EP-AAT was developed. The EP-AAT is an adapted version of the substance AAT used in our previous studies (34, 43). It consisted of one appetitive, one positive, and one negative block to prime emotional context (appetitive, positive, and negative) with 1 min breaks in between. Block order was counterbalanced across participants. To prime an emotional context, alcohol-related pictures were mixed with either appetitive, positive, or negative pictures (Figure 1). Consequently, four pictures types (alcohol, appetitive, positive, and negative) were included in the task. A total of 10 unique alcohol-related pictures of young adults drinking alcohol in a social setting were shown in all 3 emotional contexts resulting in a total of 30 alcohol pictures. In line with the standard alcohol AAT, the appetitive block contained 10 visually matched appetitive pictures of young adults drinking non-alcoholic beverages as control pictures (35, 44). The positive block contained 10 pictures of positive situations (i.e., young adults cheering, laughing, or playing), and the negative block contained 10 pictures of negative situations (i.e., young adults crying or fighting). The pictures were rotated 3° to the left or right, and participants had to pull (approach) or push (avoid) a joystick in response to the rotation direction of the picture as quickly as possible. Half of the participants had to push pictures rotated to the left and pull pictures rotated to the right, whereas the other half of the participants were given opposite instructions. The EP-AAT contains a zooming mechanism (45): upon pulling the joystick, the picture size increases on the screen (creating a sense of approach), and upon pushing the joystick, the picture size decreases on the screen (creating a sense of avoidance). In each block, each picture was presented twice in pull and twice in push format, resulting in 80 trials per block. Thus, within each block participants pushed and pulled both alcohol images and pictures from the contrast category (i.e., either appetitive, positive, or negative) equally often. Trials were presented in pseudorandom order with no more than three similar picture formats and picture types in a row. The picture remained on the screen until the push or pull response was complete, at which point the reaction time was logged. After a full response was made, a feedback screen appeared for 1000 ms. This was a blank screen when the response was correct and a central red cross when the response was incorrect. Error trials were repeated until performed correctly. The next trial started immediately after the feedback screen. Time taken to complete the task was on average 10 min. To validate emotional valence of the pictures, each participant rated a subset of 15 pictures during a picture-rating task that was performed after the EP-AAT. Valence was assessed with a visual analog scale ranging from −100 (very negative) to 100 (very positive) asking “How positive or negative do you rate this picture?”

### Procedure

Testing took place at the Erasmus Behavioral Lab. All participants first read an information letter describing the goal and procedure of the study, including a statement on guaranteed confidentiality. After signing informed consent, participants filled out questionnaires on demographic variables, alcohol craving, and substance use and alcohol use motives. Participants then performed the EP-AAT followed by the picture-rating task. Finally, participants completed the alcohol craving questionnaire a second time. Participants were reimbursed 7 Euros for their participation or received participant credits as part of psychology bachelors program of the Erasmus University.

### Data preparation and statistical analyses

Independent sample *t*-tests were used to compare demographics and scores on questionnaires between groups. Reaction time data in the EP-AAT were corrected for outliers by removing reaction times below 200 ms, above 2000 ms, and more than 3 standard deviations from the individual participant’s mean RT. Error trials were also removed. For each participant, alcohol bias-scores were calculated in each emotional context (i.e., alcohol-appetitive, alcohol-positive, alcohol-negative) by subtracting the mean approach reaction time from the mean avoid reaction time. In addition, bias-scores were calculated for the other picture types: one appetitive bias-score, one positive bias-score, and one negative bias-score. A positive score indicates faster approach compared to avoidance (approach bias). Reliability of the EP-AAT was investigated by calculating Cronbach’s *α* for each bias-score with the individual bias-scores per picture. Internal reliability of the alcohol-appetitive bias (10 items, Cronbach’s *α* = 0.38), alcohol-positive bias (10 items, Cronbach’s *α* = 0.46), alcohol-negative bias (10 items, Cronbach’s *α* = 0.43), appetitive bias (10 items, Cronbach’s *α* = 0.44), positive bias (10 items, Cronbach’s *α* = 0.20), and negative bias (10 items, Cronbach’s *α* = 0.48) was fairly poor (46) but not unusual for implicit reaction time tests (47, 48).

Statistical analyses were performed in SPSS for Windows (v.20). EP-AAT bias-scores were analyzed in two repeated measures ANOVA (RM-ANOVA). Similarly as in Wiers et al. (35), the first RM-ANOVA included bias-scores for all four picture types (i.e., alcohol-appetitive, appetitive, positive, and negative contexts) and was performed to investigate whether approach/avoid tendencies differed between the four picture types and groups. In this analysis, Picture Type was included as a four-level within-subject factor (alcohol-appetitive, appetitive, positive, and negative) and Group (occasional versus heavy drinkers) as a two-level between-subject factor. The original alcohol AAT included alcohol, other appetitive, positive, and negative pictures [see Ref. (35)]. In later versions of the AAT, alcohol pictures were combined with appetitive control images only [i.e., (44, 49)] or with appetitive control images and abstract images [i.e., (50)]. To allow a comparison with previous alcohol AAT studies but avoid the influence of the positive and negative pictures on the expression of the alcohol bias, the alcohol bias-score in the appetitive condition was compared with the bias-scores for appetitive, positive, and negative pictures. A second RM-ANOVA included bias-scores for alcohol in the three different contexts (i.e., alcohol-appetitive, alcohol-positive, and alcohol-negative) and was performed to investigate the role of primed emotional context on alcohol bias in occasional and heavy drinkers. In these analyses, Context was included as a three-level within-subject factor (alcohol-appetitive, alcohol-positive and alcohol-negative) and Group (occasional versus heavy drinkers) as a two-level between-subject factor. A third RM-ANOVA was performed to investigate whether the valence ratings differed between the picture types and groups. Again, Picture Type was included as a four-level within-subject factor (alcohol-appetitive, appetitive, positive, and negative) and Group (occasional versus heavy drinkers) as a two-level between-subject factor. Greenhouse–Geisser-adjusted *F*- and *p*-values are reported when sphericity cannot be assumed. Significant main and interaction effects were further investigated with *t*-tests Bonferroni corrected for multiple comparisons. Moreover, one-sample *t*-tests were used to investigate whether the bias-scores and the valence ratings deviated from zero.

Pearson correlations were computed to investigate the univariate associations between the alcohol bias-scores in the different contexts and measures of alcohol use, craving and coping, and enhancement drinking motives. Furthermore, regression analyses were performed to investigate the role of coping motives, enhancement motives, and alcohol problems (AUDIT) in the expression of the alcohol-approach bias in a negative and positive emotional context relative to the alcohol bias in the appetitive control condition. With this approach, we specifically investigated the extent to which drinking motives and alcohol problems can explain the change in alcohol bias-score from an appetitive context to a negative or positive emotional context.

## Results

### Sample characteristics

Groups did not differ on age (range 18–30) and gender distribution. Heavy drinkers compared to occasional drinkers scored higher on all drinking measures, pre-test craving, and post-test craving. Lifetime cannabis and illicit substance use did not differ between groups (see Table 1 for details). Heavy drinkers compared to occasional drinkers also scored higher on all drinking motives (DMQ-S social, coping, excitement, and conformity).

**Table 1:** **Sample characteristics**.

	Heavy drinkers	Occasional drinkers	*p*
*N* (female)	66 (26)	50 (30)	0.485
Age	21.5 (2.14)	21.9 (2.5)	0.442
Alcohol use, related problems (AUDIT)	12.5 (3.9)	4.3 (1.9)	**<0.001**
Alcohol use, last 10 days (TLFB; # drinks)	29.1 (17.7)	6.9 (7.1)	**<0.001**
Alcohol use, age first time	13.5 (2.0)	16.3 (12.3)	**0.022**
Alcohol use, age first time drunk	15.1 (1.4)	17.1 (2.3)^a^	**<0.001**
Alcohol use, age first binge episode	15.0 (1.3)	16.8 (1.9)^b^	**<0.001**
Alcohol use, drunk last month (#)	5.6 (2.7)	1.9 (1.0)	**<0.001**
Cigarette smoking ever (%)	77	56	**0.015**
Lifetime cigarette use (#)	18,924.6 (125,953.5)	384.4 (1708.6)	0.301
Lifetime cannabis use (#)	137.0 (406.9)	28.8 (112.5)	0.071
Lifetime illicit substance use (#)	5.6 (14.5)	2.9 (10.4)	0.267
Alcohol craving (DAQ), pre-test	23.0 (9.0)	17.8 (7.2)	**0.001**
Alcohol craving (DAQ), post-test	22.7 (9.4)	18.3 (8.0)	**0.009**
Drinking motives (DMQ-R), social	18.1 (3.0)	13.8 (5.0)	**<0.001**
Drinking motives (DMQ-R), coping	8.6 (3.1)	6.6 (2.0)	**<0.001**
Drinking motives (DMQ-R), enhancement	14.2 (4.2)	11.1 (4.7)	**<0.001**
Drinking motives (DMQ-R), conformity	6.4 (1.9)	5.7 (1.3)	**0.016**

Mean (SD); significant *p*-values for group differences are bold;

^a^*n* = 37 and

*^b^*n* = 45; AUDIT, Alcohol Use Disorder Identification Test; heavy drinkers, AUDIT ≥8; occasional drinkers, AUDIT <8; TLFB, timeline follow back; DAQ, Desires for Alcohol Questionnaire; DMQ-R, Drinking Motives Questionnaire – Revised*.

### Valence ratings

**Figure 2:**
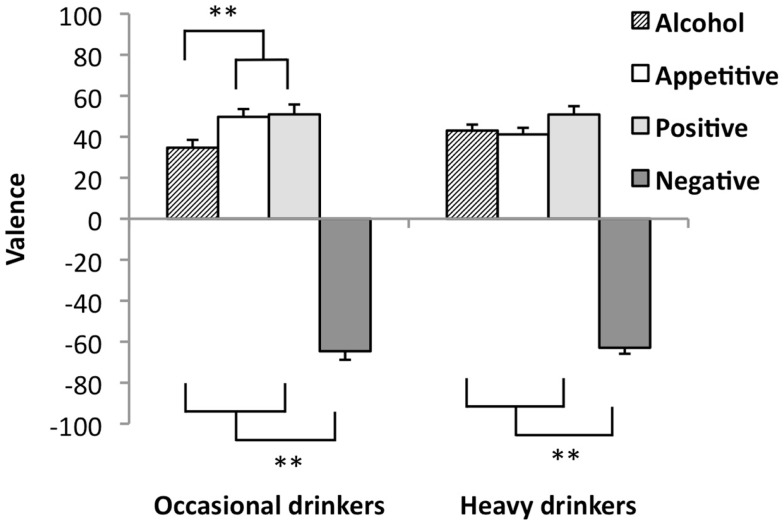
**Valence ratings of EP-AAT pictures for heavy (*n* = 62) and occasional drinkers (*n* = 48)**. Pictures were rated on a scale from −100 to 100. Mean valence scores are shown with standard error bars; **p < 0.001.

Unfortunately, valence ratings were missing from two occasional and four heavy drinkers. Valence ratings were analyzed in the remaining sample of 48 occasional drinkers and 62 heavy drinkers. A main effect for Picture Type was found for the valence rating in the Picture Type (alcohol-appetitive, appetitive, positive, negative) × Group (occasional, heavy drinkers) RM-ANOVA, *F*(3,106) = 270.37, *p* < 0.001. Moreover, the interaction between Picture Type and Group was significant, *F*(3,106) = 4.24, *p* = 0.007. *Post hoc* analyses indicated that within the group of occasional drinkers, alcohol pictures were rated less positive than the appetitive and the positive pictures (*p* < 0.001), but more positive than the negative pictures (*p* = 0.001). Appetitive pictures were rated as positive as the positive pictures. Within the group of heavy drinkers, rating of the alcohol and appetitive and positive pictures did not differ. Alcohol, appetitive, and positive pictures were rated more positive than the negative pictures (*p* < 0.001). One sample *t*-tests indicated that the alcohol, appetitive, and positive pictures were rated positive (*p* < 0.001), whereas the negative pictures were rated negative (*p* < 0.001) in both groups. See Figure 2 for valence ratings of EP-AAT pictures per group.

### Emotional prime approach avoidance task

**Figure 3:**
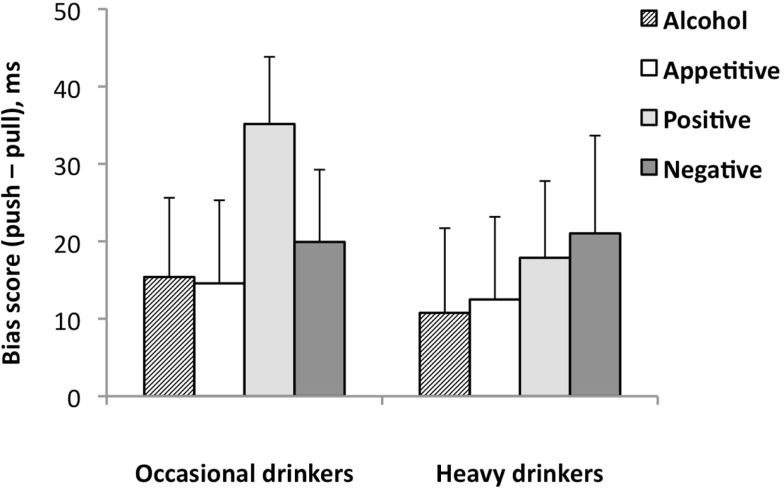
**Emotional prime approach avoidance task bias-scores for alcohol, appetitive, positive, and negative pictures in heavy drinkers (*n* = 65) and occasional drinkers (*n* = 50)**. Mean bias-scores are shown with standard error bars.

**Figure 4:**
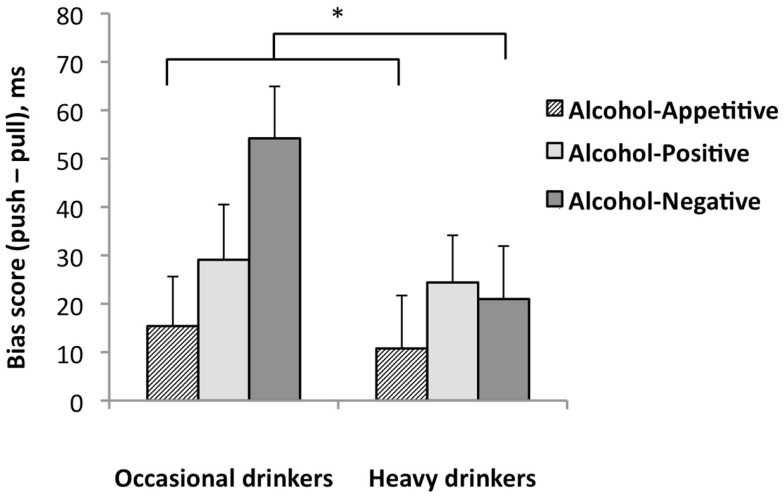
**Emotional prime approach avoidance task bias-scores for alcohol in the appetitive, positive, and negative contexts in heavy drinkers (*n* = 65) and occasional drinkers (*n* = 50)**. Mean bias-scores are shown with standard error bars; *p < 0.05.

One heavy drinker made 60% errors during the EP-AAT and was therefore excluded from further analyses. EP-AAT performance of the remaining sample (*n* = 115) was on average 95% correct (min = 85%), with no group differences in accuracy and mean RT. The Picture Type (alcohol-appetitive, appetitive, positive, negative) × Group (occasional, heavy drinkers) RM-ANOVA did not reveal a main or interaction effect of Picture Type and Group (all *p*s > 0.44), suggesting that the different picture types were similarly approached in both groups. A main effect for Context was found for alcohol bias-scores in the Context (alcohol-appetitive, alcohol-positive, alcohol-negative) × Group (occasional, heavy drinkers) RM-ANOVA, *F*(2,112) = 3.33, *p* = 0.039. No main or interaction effects of Group were found. Follow-up *t*-tests indicated that the alcohol-approach bias in the negative context was significantly larger than the alcohol-approach bias in the appetitive context, *p* = 0.034. Separate *post hoc* paired *t*-tests for the pull and push mean reaction times showed that participants were slower to avoid alcohol (i.e., mean reaction times for push were longer) in the negative context than in the appetitive context, *t*(114) = 2.9, *p* < 0.01, whereas there was no context effect in the approach condition (i.e., mean reaction times for pushing were not different). These findings may suggest that alcohol-drinking students had difficulties avoiding alcohol in a negative emotional context. In addition, *post hoc t*-tests comparing overall RTs during the negative, positive, and appetitive contexts indicated that RTs were generally slower in the negative compared to the appetitive context (mean difference 27 ms, *p* < 0.001). One sample *t*-test across the groups indicated that the participants had an alcohol-approach bias in all contexts (alcohol-appetitive *p* = 0.049, alcohol-positive *p* = 0.001, alcohol-negative *p* < 0.001). Moreover, there was a significant approach bias for negative (*p* = 0.024) and positive (*p* < 0.001), but not for the appetitive images (*p* = 0.081). See Table 2 and Figures 3 and 4 for mean EP-AAT bias-scores per group.

**Table 2:** **Emotional prime approach avoidance task reaction times**.

	Pull		Push	
	Mean	SD	Mean	SD
Alcohol-appetitive	771.0	124.1	785.8	118.9
Alcohol-positive	769.9	124.7	796.4	129.5
Alcohol-negative	775.3	119.5	810.7	130.4
Appetitive	761.3	125.2	774.7	116.8
Positive	782.0	130.1	807.4	128.5
Negative	799.2	126.1	817.3	124.6

*SD, standard deviation*.

### Correlations

The alcohol bias in the appetitive, positive, and negative contexts did not significantly correlate with measures of alcohol use, craving or coping, and enhancement drinking motives. There were also no significant correlations when only the heavy drinkers were included in the analyses. Coping and enhancement drinking motives were significantly correlated (*r* = 0.47, *p* < 0.001) and also correlated with measures of alcohol use, problems, and craving: first time drunk (coping: *r* = −0.22, *p* = 0.025; enhancement: *r* = −0.23, *p* = 0.019), drunk last month (coping: *r* = 0.33, *p* < 0.001; enhancement: *r* = 0.42, *p* < 0.001), alcohol use last 10 days (TLFB; coping: *r* = 0.30, *p* < 0.001; enhancement: *r* = 0.35, *p* < 0.001), alcohol-related problems (AUDIT; coping: *r* = 0.48, *p* < 0.001; enhancement: *r* = 0.46, *p* < 0.001), pre-test craving (DAQ; coping: *r* = 0.52, *p* < 0.001; enhancement: *r* = 0.42, *p* < 0.001), and post-test craving (DAQ; coping: *r* = 0.48, *p* < 0.001; enhancement: *r* = 0.46, *p* < 0.001). Given that the negative context effect was driven by slower avoidance in the negative compared to the appetitive context, we performed additional correlational analyses to investigate if this slowing of avoidance (RT push alcohol-negative minus RT push alcohol-appetitive) was related to measures of alcohol use, craving, and drinking motives. The slowing of avoidance did, however, not significantly correlate with any of these measures.

### Relationship of drinking motives and alcohol bias in a primed emotional context

In order to assess if individual differences in drinking motives and alcohol-related problems increased the alcohol bias in the negative emotional context, a hierarchical multiple regression analysis was performed. The alcohol bias-score in the appetitive (control) context was entered in the first step and the DMQ-R coping, DMQ-R enhancement, and AUDIT scores in the second step. Preliminary analyses indicated no violation of the assumption of normality, linearity, multicollinearity, and homoscedasticity (maximum Cook’s distance = 0.12, maximum standardized residual = 3.00). The final model explained 19% (adjusted 16%) of the variance in alcohol-negative bias-score, *F*(4,110) = 6.21, *p* < 0.001. The alcohol-appetitive bias-score was a significant predictor of the alcohol-negative bias-score (11% explained variance, *p* < 0.001). Drinking motives and alcohol use-related problems explained an additional 8% of the variance in alcohol bias-scores in the negative context, *F*_change_(3,110) = 3.37, *p* = 0.021. Enhancement motives (β = 0.24, *p* = 0.024) and AUDIT scores (β = −0.29, *p* = 0.005), but not coping motives (β = 0.01, *p* = 0.93), uniquely explained variance in the alcohol bias-score in the negative context. Higher enhancement motives and lower alcohol use-related problems were related to an increase in alcohol bias-score in the negative emotional context.

A second parallel hierarchical multiple regression analysis was performed to investigate if individual differences in drinking motives and alcohol use-related problems increased the alcohol bias in the positive emotional context. The alcohol bias-score in the appetitive (control) context was entered in the first step and the DMQ-R coping, DMQ-R enhancement, and AUDIT scores in the second step. Preliminary analyses indicated no violation of the assumption of normality, linearity, multicollinearity, and homoscedasticity (maximum Cook’s distance = 0.18, maximum standardized residual = 2.90). The final model explained 13% (adjusted 10%) of the variance in alcohol-positive bias-score, *F*(4,110) = 4.12, *p* = 0.004. The alcohol-appetitive bias-score was a significant predictor of the alcohol-positive bias-score (13% explained variance, *p* < 0.001), whereas drinking motives and alcohol use-related problems were not. Enhancement motives, coping motives, and alcohol use-related problems were therefore not related to an increase in alcohol bias-score in the positive emotional context.

## Discussion

The present study tested whether implicitly activated alcohol-approach action tendencies could be distinguished based on a primed emotional context (positive, negative, neutral appetitive) and whether negative and positive reinforcement motives to drink could be assessed in an indirect or implicit way using this method. The findings showed that the primed emotional context influenced the approach bias in both occasional and heavy drinkers. More specifically, the alcohol-approach bias increased in the negative context relative to the appetitive (non-alcoholic) control context. This context effect was driven by slower avoidance (not faster approach) suggesting that the avoidance of alcohol-related cues may generally be more difficult in a negative emotional context. Perhaps surprisingly, this change in alcohol-approach bias in the negative context was positively related to explicit enhancement drinking motives (but not coping motives) and negatively related to alcohol use-related problems. In contrast to our hypotheses, the alcohol-approach bias in the positive contexts did not interact with drinking level and explicit enhancement motives. In addition, the expected stronger approach bias for alcohol in heavy drinkers versus occasional drinkers was not found. These findings are discussed in the context of a larger literature on implicit and explicit cognitive processes, in relation to alcohol use.

The finding of slower avoidance of alcohol-related cues in a negative context in all drinking students may suggest that the incentive value of context can influence the tendency to approach or avoid alcohol. More specifically, it may be that a negative primed context could act as a cue hampering the ability to avoid alcohol. The fact that we observed a modulation of the alcohol-approach bias by the negative and not the positive primed context may be explained by the subjective valence ratings of the pictures. Negative pictures were rated more negatively than all the other picture types, whereas no differences in subjective ratings were found between appetitive, positive, and alcohol pictures.

An alternative explanation could be that arousal may modulate action tendencies, not valence. Negative pictures are generally rated as more arousing (51) and regardless of valence, arousal has been related to emotional interference during an emotional Stroop task (52). Comparing overall RTs (averaged across picture type and push/pull response) during the negative, positive, and appetitive contexts indicated that RTs were generally slower in the negative compared to the appetitive context. This effect was found regardless of the direction of response. It may be that the arousing contents of the negative pictures captured attention such that it slowed down the detection of the orientation of the pictures. Even though this effect was seen for both pull and push responses, it may be more pronounced for avoidance as push responses were generally slower than pull responses. Unfortunately, the current design does not allow us to differentiate between arousal and valence effects on action tendencies. However, this could be an interesting issue to explore in future studies. Moreover, if this assumption holds true, it suggests the relevance of general arousal effects on attention which may not be specific to alcohol pictures. The inclusion of a pure neutral image category (e.g., office stationery) instead of appetitive images is thereby recommended.

We observed that explicit enhancement drinking motives were related to increased alcohol-approach bias in the negative context, not in the positive context. Building further on the general arousal idea, this could suggest that arousal may influence appetitive alcohol responses in individuals with strong enhancement drinking motives (which have to do with drinking in a positive arousing context). Unexpectedly, the effect of the negative context was smaller in drinkers with higher levels of alcohol-related problems. Alcohol-approach tendencies may become more habitual over the course of alcohol use toward dependence (37), suggesting that specific emotional contexts may become less of an influence on the expression of alcohol action tendencies. Yet Ostafin and Brooks (20) showed that a negative mood induction is capable to increasing alcohol-approach associations in coping drinkers. The EP-AAT was not designed to induce a negative or positive mood, and the subtle context priming in the current study may therefore have been too weak. Automatic activation of appetitive responses for alcohol may only be triggered in coping drinkers after priming of the associated mood state. One possibility to further explore the relationship between alcohol-approach tendencies, emotional context, and drinking motives is to employ a strong positive and negative mood induction paradigm.

The finding that heavy drinkers, relative to occasional drinkers, did not show a stronger approach bias for alcohol across different primed emotional contexts was unexpected. However, it should be noted that results in previous studies using indirect tests like the AAT were not very consistent (37), which may be attributed to differences in methodology and sample characteristics. In addition, a recent discussion concerning reaction time measures of implicit constructs such as approach and attentional biases raised the issue of limited internal reliability of these reaction time measures (47, 48, 53). Since then, a number of studies (including the current one) have reported internal reliability of reaction time measures. Unfortunately, the internal reliabilities of bias-scores in the current study were relative low, although comparable to other studies (34, 43, 54). In the EP-AAT, we included pictures of relative complex social scenes, which may in part explain the poor reliability (47, 48). This relative low internal reliability of bias-scores is a limitation of the current study and studies using implicit measures in general. Developing reaction time measures with higher internal reliably for implicit constructs such as approach bias is of major importance and should be a priority in future research.

An alternative explanation of the negative findings is that the student sample did not score high enough on coping motives (see Table 1, students primarily drink for social and enhancement reasons). However, a *post hoc* analysis in which coping scores were compared for alcohol approach biases in the negative context by quartile did not show differences in coping scores for the different alcohol approach ranks, thereby providing additional support for the lack of a moderating effect of coping motives on alcohol approach biases in the negative context. One remaining possibility is that automatic activation of approach tendencies in a negative context only occurs at a certain threshold of coping motives, which has not been reached in the current student sample. In addition, our drinking students may represent a group with relatively high control capacities such as working memory, which could have resulted in increased control over reactions to alcohol stimuli (7–10), thereby reducing possible differences between approach tendencies across the different primed contexts.

As another alternative explanation for the current findings, it is possible that the observed increase in alcohol-approach bias in a negative context is a general effect across different types of drinkers, including occasional and heavy drinkers. This could imply that negative reinforcement plays a role in different types of drinking behavior, a finding that contradicts contemporary addiction theory in which negative reinforcement is mainly important after the stage of binge drinking (55).

In conclusion, the current study investigated whether an alcohol-approach bias in occasional and heavy drinkers was modulated by negative and positive emotional primes. While a stronger alcohol-approach bias in heavy drinkers could not be replicated, the current findings suggest that both occasional and heavy drinkers have a selective difficulty to avoid alcohol-related cues in a negative emotional context. This change in alcohol-approach bias was positively related to explicit enhancement drinking motives and negatively related to alcohol-related problems. Negative reinforcement may therefore be involved in different types of drinking patterns. However, the influence of emotional context on alcohol-related action tendencies may become smaller when alcohol use becomes more problematic, which is in line with habit accounts of addiction.

## Author Contributions

All authors contributed to the study design. Janna Cousijn and Maartje Luijten coordinated data collection and performed data analyses. All authors contributed to interpretation of data and drafting the manuscript. All authors approved publication of the manuscript and agreed to be accountable for all aspects of the work.

## Conflict of Interest Statement

Janna Cousijn and Maartje Luijten declared no conflict of interest. Reinout W. Wiers gave a paid talk for Lundbeck pharmaceutical company, and Reinout W. Wiers was co-applicant in two awarded grants from ERAB (The European Foundation for Alcohol Research), which is an independent foundation that awards alcohol-related research after an independent scientific evaluation (peer reviewed), with guarantee of completely independent scientific expression (in accordance with the Dublin principles), http://www.api.or.at/sp/alcoholpolicy%20dokumente/dublinpriciples.pdf. Reinout W. Wiers was also involved in the ERAB Underage Drinking Report (2012), which was also done in accordance with the Dublin principles.
